# Chikungunya virus nonstructural protein 1 is a versatile RNA capping and decapping enzyme

**DOI:** 10.1016/j.jbc.2023.105415

**Published:** 2023-10-31

**Authors:** Michelle Cheok Yien Law, Kuo Zhang, Yaw Bia Tan, Trinh Mai Nguyen, Dahai Luo

**Affiliations:** 1 Lee Kong Chian School of Medicine, Nanyang Technological University, Singapore, Singapore; 2 NTU Institute of Structural Biology, Nanyang Technological University, Singapore, Singapore; 3 National Centre for Infectious Diseases, Singapore, Singapore

**Keywords:** 5' end cap-0, positive-sense RNA virus, mRNA, interferon, decapping

## Abstract

Chikungunya virus (CHIKV) nonstructural protein 1 (nsP1) contains both the N7-guanine methyltransferase and guanylyltransferase activities and catalyzes the 5′ end cap formation of viral RNAs. To further understand its catalytic activity and role in virus–host interaction, we demonstrate that purified recombinant CHIKV nsP1 can reverse the guanylyl transfer reaction and remove the m^7^GMP from a variety of capped RNA substrates including host mRNAs. We then provide the structural basis of this function with a high-resolution cryo-EM structure of nsP1 in complex with the unconventional cap-1 substrate RNA m^7^GpppA_m_U. We show that the 5′ppRNA species generated by decapping can trigger retinoic acid–inducible gene I–mediated interferon response. We further demonstrate that the decapping activity is conserved among the alphaviral nsP1s. To our knowledge, this is a new mechanism through which alphaviruses activate the antiviral immune response. This decapping activity could promote cellular mRNA degradation and facilitate viral gene expression, which is functionally analogous to the cap-snatching mechanism by influenza virus.

Alphaviruses have their own capping mechanisms and employ different viral enzymes to facilitate this process, which makes them an attractive target for antiviral drug development. They are positive-sense ssRNA viruses that replicate in the cytoplasm where the genome contains two ORFs. The first encodes the nonstructural proteins nsP1-4, while the second under the control of the subgenomic promoter encodes the structural proteins. Both RNA transcripts are capped with nsP1-2 capping machinery where nsP1 contains both N7-guanine methyltransferase (MTase) and guanylyltransferase (GTase) activities (1, 2) while nsP2 contains the N-terminal RTPase/helicase domain which hydrolyzes the β–γ phosphate bond at the RNA 5′ triphosphate end (3, 4). Cryo-EM structures of the chikungunya virus (CHIKV) nsP1 have been reported recently (5, 6). Twelve copies of nsP1s organize into a dodecameric ring structure with the bifunctional MTase/GTase catalytic domain forming the upper ring, while the lower ring is responsible for oligomerization and membrane association. nsP1 catalyzes the 7-methylguanosine (m^7^G) cap (Cap-0) formation differently from the eukaryotic mRNA capping machinery: the methyl group is added to the N7 of GTP by MTase, following which the m^7^Gp moiety is transferred to the 5′ diphosphate end of viral RNA by GTase (1, 2, 7, 8).

In eukaryotes, the decapping process is the reverse of the capping reaction as part of the mRNA degradation pathway to regulate mRNA gene expression where the 5′ cap is removed where thereby allowing targeting by exoribonuclease in the 5′-3′ pathway. Uncapped RNAs such as viral transcripts are targets for degradation by host enzymes and may trigger antiviral innate immune responses. The host antiviral response is activated through the expression of interferon-stimulated genes (ISGs), which target viral RNAs through sensing by cytoplasmic pattern recognition receptors such as retinoic acid–inducible gene I (RIG-I) and MDA5. They detect dsRNA viral replicative intermediates, molecular features associated with viral genomes, or the absence of features associated with host mRNAs, resulting in degradation and repression of viral RNA expression. As such, viruses have several strategies to acquire the 5′ cap structure (9). They can synthesize cap structures using their own or cellular capping processes or steal cap structures from cellular mRNAs (7).

Some viruses lack capping machinery including L-A virus, a dsRNA virus that infects the yeast *Saccharomyces cerevisiae*. In this scenario, decapping can function to overcome the disadvantages of uncapped viral transcripts as the L-A capsid Gag protein can form a covalent bond between m^7^Gp and H154, resulting in the removal of mRNA cap from cellular mRNA. This results in increased viral mRNA expression through the generation of capless cellular mRNAs, which act as decoys for the cellular mRNA decay system (10). Another prototypic viral decapping enzyme D10 in the vaccinia virus contains a Nudix hydrolase domain that cleaves to form diphosphate product m^7^Gpp (11). Decapping by D10 serves as a mechanism for controlling host and viral gene expression as it fails to discriminate between viral and cellular mRNAs, leading to the degradation of both viral and host RNAs. Decapping activities of viral enzymes seem to be a common mechanism to modulate the host mRNA metabolism and facilitate virus replication.

Here, we demonstrate the decapping activity of CHIKV nsP1, through *in vitro* and cell-based assays. The high-resolution cryo-EM structure of CHIKV nsP1 in complex with a short, capped RNA, m^7^GpppA_m_U, which is unlike the native Cap-0 structure formed by the alphavirus capping machinery suggests that decapping can target host cellular mRNAs. In addition, we demonstrate how decapping of viral RNAs activates RIG-I–mediated type I interferon (IFN) signaling through the generation of 5′ diphosphate RNA by-products as a result of CHIKV nsP1–mediated decapping.

## Results

### CHIKV nsP1 is a versatile capping/decapping enzyme

**Figure 1:**
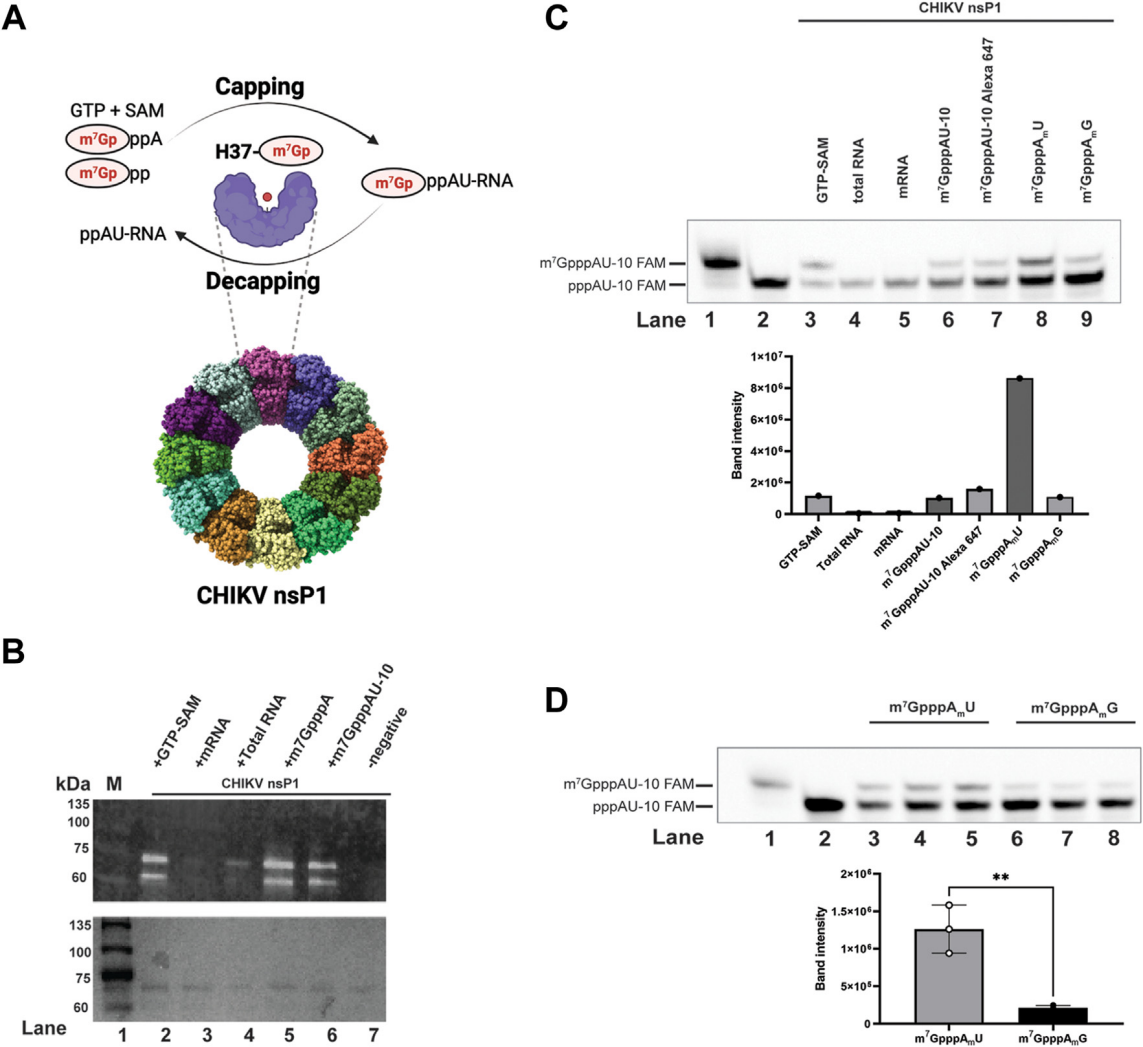
**Capping/decapping activity with recombinant CHIKV nsP1 with different capped RNAs.***A*, overview of capping and decapping *in vitro*. Capping occurs when m^7^Gp is removed from cap donor to form the covalent intermediate m^7^Gp-nsP1 and the m^7^Gp is then transferred to a suitable cap substrate to form capped RNA. In decapping, m^7^Gp is removed from capped RNA, resulting into ppRNA. *B*, decapping activity of recombinant nsP1 by detection of m^7^Gp-nsP1 intermediate through immunoblotting after incubation with GTP/SAM (*lane 2*), mRNA (*lane 3*), total RNA (*lane 4*), m^7^GpppA (*lane 5*), m^7^GpppAU-10 (*lane 6*), and protein only (*lane 7*). Marker is denoted as M with the corresponding SDS-PAGE gel below. *C*, *above:* capping activity of recombinant nsP1 incubated with GTP-SAM (*lane 3*), 150 ng of total RNA (*lane 4*), and 150 ng of mRNA (*lane 5*) extracted from mammalian Expi293 cells, m^7^GpppAU-10 (*lane 6*), m^7^GpppAU-10 Alexa 647 (*lane 7*) at 1 mM, m^7^GpppA_m_U (*lane 8*), and m^7^GpppA_m_G (*lane 9*) both at 5 mM. The capped RNA substrate m^7^GpppAU-10 FAM (*lane 1*) and uncapped RNA substrate pppAU-10 FAM (*lane 2*) as references visualized by UREA PAGE gel. *Below:* the bands in each lane of the gel image were analyzed and measured where the amount of capped RNA is proportional to the band intensity as plotted to compare the amount of capped RNA product in each lane. *D*, above: capping activity of recombinant nsP1 with both m^7^GpppA_m_U in *lanes 3 to 5* (5 mM) and m^7^GpppA_m_G in *lanes 5 to 7* (5 mM) as cap donor substrate in decreasing concentrations of nsP1 0.5 μM, 0.2 μM, and 0.1 μM. *Lanes 1 and 2* are references for m^7^GpppAU-10 FAM and pppAU-10 FAM without nsP1 respectively visualized by UREA PAGE gel. *Below:* the bands in each lane of the gel image were analyzed and measured where the amount of capped RNA is proportional to the band intensity as plotted ∗∗ <0.01, unpaired *t* test *p*-value <0.01 95% CI −1565160 to −534036. CHIKV, chikungunya virus; m^7^G, 7-methylguanosine; nsP, nonstructural protein.

To examine if the RNA 5′end capping reaction may be reversible, we measured CHIKV nsP1 capping/decapping activity (Fig. 1*A*). First, CHIKV nsP1 was able to snatch the m7Gp moiety from several biological and chemical sources, such as total RNA and mRNA extracted from Expi293 cells, m^7^GpppA, and capped RNA m^7^GpppAU-10. The formation of m^7^Gp-nsP1 covalent intermediate was detected by immunoblotting of m^7^Gp moiety on nsP1 protein (Fig. 1*B*). Next, we show that the m^7^Gp-nsP1 covalent intermediate could successfully transfer m^7^G to a suitable RNA substrate (5′ pppAU-10 RNA oligo) and result in capped RNA product, despite varying efficiency (Fig. 1*C* above). Cap-0 donors such as m^7^GpppAU-10, m^7^GpppAU-10 Alexa 647 (Fig. 1*C* lane 6 and 7), as well as short-capped RNAs m^7^GpppA_m_U and m^7^GpppA_m_G (Fig. 1*C* lane 8 and 9) could promote the capping of 5′ pppAU-10 RNA oligo. Using 150 ng of total RNA and mRNA each did not (Fig. 1*C*, lane 4 and 5) result in detectable capping, likely due to the heterogeneity of RNA length, low concentration or both. We further quantified the capping reactions by analyzing the band intensity, which is proportional to the amount of capped RNAs product formed (Fig. 1*C* below). Previous work demonstrated sequence preference for the capping (12) which may also apply in the reverse reaction as m^7^GpppA_m_U (Fig. 1*D*, lane 3–5) is preferred over m^7^GpppA_m_G (Fig. 1*D*, above lane 6–8). There is more capped RNA product formation when m^7^GpppA_m_U is the donor (Fig. 1*D* below) (unpaired *t* test *p*-value <0.01 95% confidence interval [CI] −1565160 to −534036). As such, the m^7^GpppA_m_ (Cap-1) structure can serve as an effective m^7^Gp donor as there is no clash for a methylated 2′ hydroxy group (2′-O-me) on the A1 ribose sugar of m^7^GpppAU-10. This suggests that nsP1 can cleave off the m^7^Gp from both Cap-0 and Cap-1 RNA, resulting in the formation of the Cap-0 RNA product. This highlights the functional versatility of nsP1 as it can potentially extract the m^7^Gp from a variety of capped RNAs.

### Decapping host mRNAs by nsP1 triggers RIG-I–mediated IFN response

**Figure 2:**
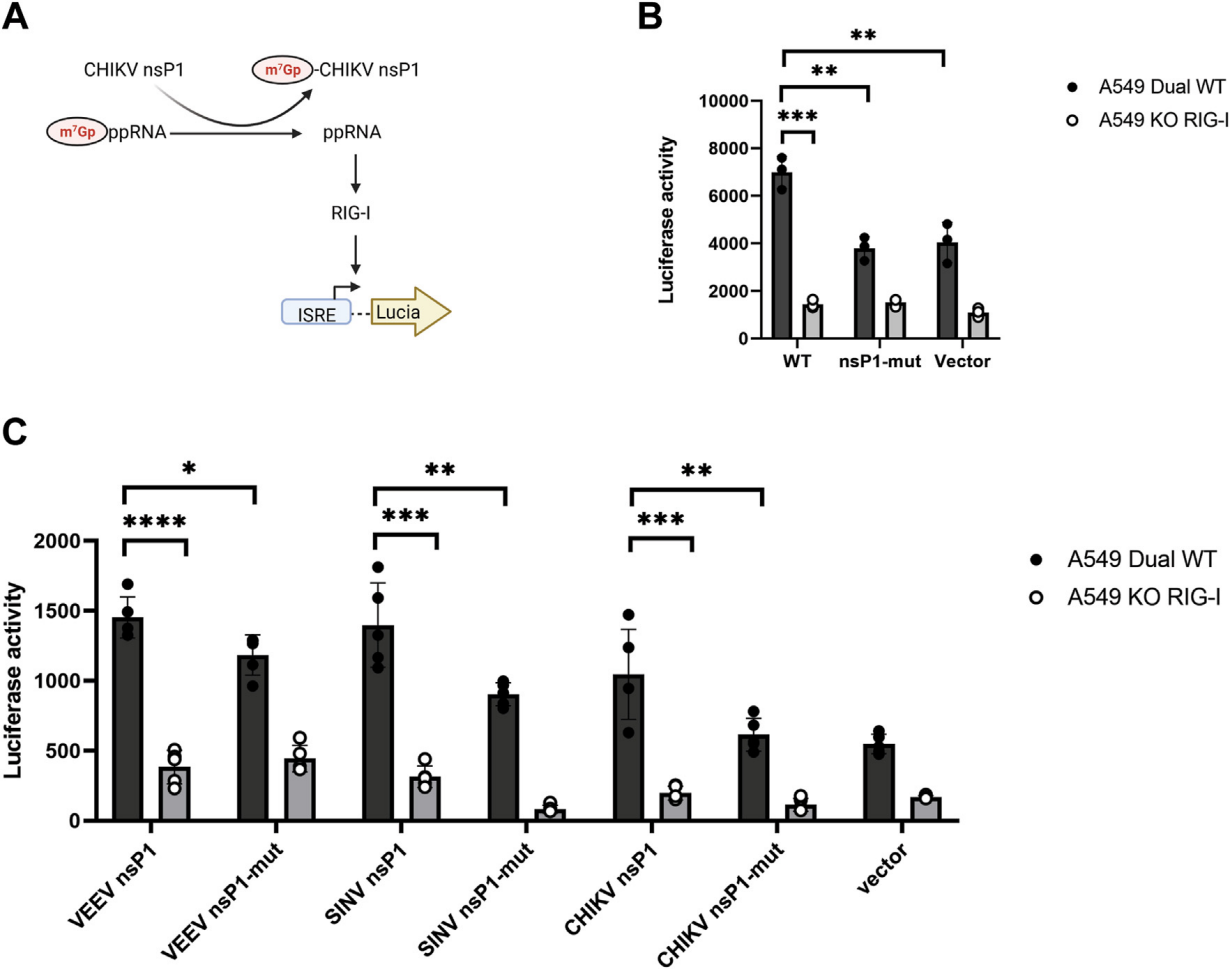
**Alphavirus nsP1 decapping activity forms ppRNA products which activates interferon pathway *via* RIG-I.***A*, hypothesized mechanism nsP1 activating retinoic acid–inducible gene I (RIG-I) mediated type I interferon signaling through the formation of 5′ diphosphorylated RNAs as a result of the decapping activity in the reporter cell lines A549 Dual, where luciferase activity indicates interferon activation. *B*, luciferase activity detected in A549 Dual or RIG-I KO cells after transfection with plasmids expressing CHIKV nsP1 wt, nsP1-mut, and control vector. Data are represented as mean ± SEM (N = 3) *C*, luciferase activity detected in A549 Dual or RIG-I KO cells after transfection with plasmids overexpression nsP1 in alphaviruses CHIKV, VEEV, and SINV wt, nsP1-mut, and control vector. Unpaired *t* test *p*-value ns > 0.05, ∗ <0.05, ∗∗ <0.01, ∗∗∗ <0.001, and ∗∗∗∗ <0.0001. Data are represented as mean ± SEM (N = 5). CHIKV, chikungunya virus; nsP, nonstructural protein; SINV, Sindbis virus; VEEV, Venezuelan Equine Encephalitis virus.

We hypothesized that the decapping activity of nsP1 could occur within host cells given there are plenty of host mRNAs. The consequence is the production of 5′ diphosphorylated RNAs, which may in turn stimulate RIG-I–mediated IFN signaling. We then tested this hypothesis by expressing CHIKV nsP1 in the reporter A549 Dual and a RIG-I KO A549-Dual KO-RIG-I cell lines. They allow monitoring of the interferon regulatory factor 3 (IRF3) pathway through the activity of ISG54 promoter-driven Lucia luciferase (Fig. 2*A*). We expressed CHIKV nsP1 WT and the catalytically inactive mutant H37A (nsP1-mut) in A549 Dual and A549-Dual KO-RIG-I cells (Fig. 2*B*). The IRF3 pathway is significantly more stimulated when nsP1 wt is expressed in A549 Dual than nsP1-mut and the vector control (unpaired *t* test *p*-value <0.01; nsP1-mut 95% CI -4543 to −1854; vector 95% CI −4670 to −1228) as well as comparing IRF3 activation in A549 Dual and KO-RIG-I when nsP1 is expressed. These results indicate that active nsP1 indeed activates type I IFN immune response. In contrast, when expressed in the KO-RIG-I cells, no significant changes to the luciferase activities could be detected. This suggests that the IFN response is mediated through the RIG-I signaling pathway.

### Decapping activity is conserved in both Old World and New World alphavirus nsP1

Alphaviral nsP1s are highly conserved (5, 6, 12) suggesting that decapping is present among other alphaviruses. We expressed nsP1 and its inactive (H37A/H39A) mutant from Venezuelan Equine Encephalitis virus (VEEV), a New World alphavirus and another closely related Old World alphavirus Sindbis virus (SINV). There was more activation of IRF3 pathway when VEEV and SINV WT was expressed compared to the inactive mut in A549 Dual cells (unpaired *t* test *p*-value <0.0189 VEEV 95% CI −480.2 to −57.37; Unpaired *t* test *p*-value <0.0076 SINV 95% CI -816.2 to −173.0). In addition, the IRF3 pathway is significantly more stimulated when wt nsP1 from both VEEV and SINV (VEEV unpaired *t* test *p*-value <0.0001; A549-Dual KO-RIG-I 95% CI −1232 to −827.6; SINV *p*-value <0.0002 A549-Dual KO-RIG-I 95% CI −1435 to −697.1) was expressed in A549 Dual compared to RIG-I KO cell line A549-Dual KO-RIG-I cells (Fig. 2*C*). Together, this suggests the presence of decapping enzymatic activity in other alphavirus nsP1s can potentially activate IFN and it is mediated by RIG-I. Interestingly, we also observed some IFN activity in the nsP1 mutants, suggesting there is also activation independent of catalytic activity.

### Decapping product 5′ppRNA activates RIG-I

**Figure 3:**
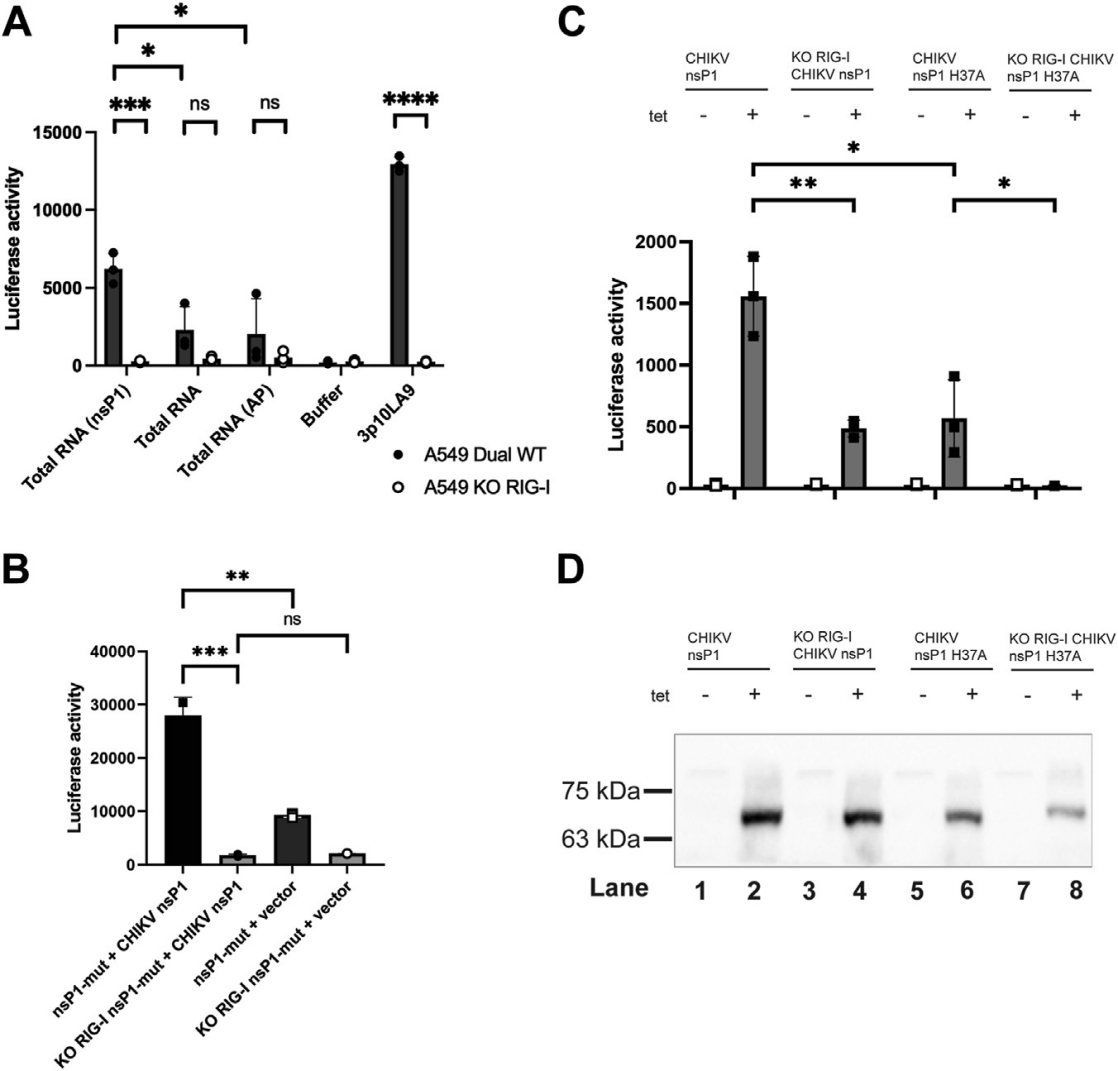
**CHIKV nsP1 enzymatic activity activates interferon mediated by RIG-I in tet-inducible expression of CHIKV nsP1 and mutant in A549 Dual and RIG-I KO cells.***A*, luciferase activity detected in A549 Dual or RIG-I KO cells after transfection with isolated RNA from Expi293 cells treated with CHIKV nsP1 or alkaline phosphatase, untreated RNA, and 3p10LA9. The hairpin RNA 3p10LA9 was used as a positive control as it specifically activates RIG-I. *B*, luciferase activity in tet-inducible expression of CHIKV nsP1-mut in A549 Dual, RIG-I KO cells with tetracycline (1 μg/ml) added and subsequently transfected with plasmid expressing CHIKV nsP1 wt and control vector. *C*, luciferase activity in tet-inducible expression of CHIKV nsP1 and nsP1-mut in A549 Dual or RIG-I KO cells with and without tetracycline (1 μg/ml) added. Unpaired *t* test *p*-value ns > 0.05, ∗ <0.05, ∗∗ <0.01, ∗∗∗ <0.001, and ∗∗∗∗ <0.0001. All data are represented as mean ± SEM (N = 3) are shown in (*A* to *C*). *D*, Western blot detection of CHIKV nsP1 in tet-inducible A549 Dual and RIG-I KO cells with and without tetracycline (1 μg/ml) at 72 h. CHIKV, chikungunya virus; nsP, nonstructural protein; RIG-I, retinoic acid–inducible gene I.

To further examine the interplay between mRNA decapping and RIG-I–mediated IFN activation, total RNA was extracted from mammalian cells and incubated with CHIKV nsP1 or alkaline phosphatase to generate either 5′ppRNA or dephosphorylated RNA, respectively. The treated RNA was extracted and transfected along with untreated RNA and the 5′ triphosphorylated hairpin RNA 3p10LA9 into A549 Dual and A549-Dual KO-RIG-I cells after which luciferase activity was measured. The IRF3 pathway is significantly more stimulated when RNA treated with nsP1 is transfected in A549 Dual compared to the RIG-I KO cell line, as well as A549 Dual transfected with untreated RNA or RNA treated with alkaline phosphatase (unpaired *t* test *p*-value <0.05; total RNA (AP) 95% CI 213.5–8176; total RNA 95% CI 1047–6813) (Fig. 3*A*). The 3p10LA9 was used as a positive control as it specifically activates RIG-I (13, 14).

Lastly, we generated A549 Dual and A549-Dual KO-RIG-I cells, which stably express CHIKV nsP1 and H37A mutant (nsP1-mut) with the induction of tetracycline. The IRF3 pathway is significantly more stimulated after tet-induced CHIKV nsP1 expression in A549 Dual compared to A549-Dual KO-RIG-I (unpaired *t* test *p*-value <0.01; 95% CI –1600 to −544.4) (Fig. 3*B*) Similar to previous experiments, the IRF3 pathway is significantly more stimulated when nsP1 is expressed in A549 Dual than nsP1-mut (unpaired *t* test *p*-value <0.05; 95% CI −991 to −270.8). In addition, there was IRF3 pathway activation when in A549 Dual tet-induced expression of CHIKV nsP1-mut cells were subsequently transfected with a plasmid containing overexpression of wt CHIKV nsP1 compared to the KO-RIG-I cells and a control vector (unpaired *t* test *p*-value <0.001; KO-RIG-I cells 95% CI −32007 to −20328; unpaired *t* test *p*-value <0.01, control cells 95% CI −24589 to −12828) (Fig. 3*C*). Together, we observe that there is both catalytic-dependent and catalytic-independent stimulation of the IFN activation and response, where the former is likely a result in 5′ppRNA production, which subsequently activates RIG-I–mediated innate immune response in the host (15).

### Structural basis of mRNA decapping by CHIKV nsP1

**Figure 4:**
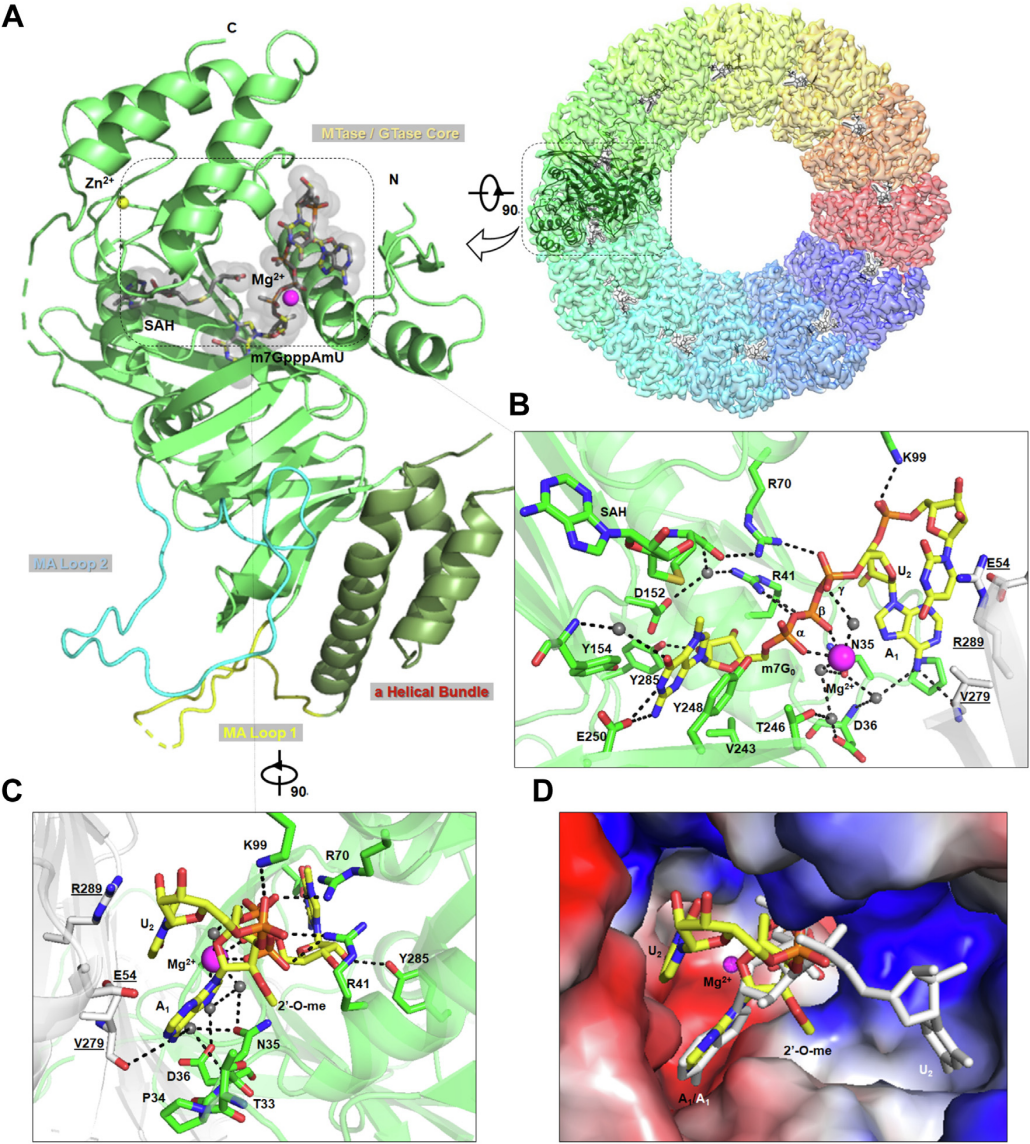
**Structural basis of RNA decapping by CHIKV nsP1.***A*, the overall structure of nsP1 with SAH and Cap-1 RNA m^7^GpppAmU. *Left*, *cartoon representation* of nsP1 monomer was colored and labeled according to the functional domains and secondary structures of nsP1, while zinc (Zn^2+^) and magnesium (Mg^2+^) ions are shown in *yellow* and *magenta*, respectively. *Right*, the nsP1 ring is shown as a semitransparent surface view with one nsP1 shown as *cartoon* and ligands are displayed as *sticks* in each of the catalytic sites. *B*–*C*, close-up view of the nsP1 monomer bound to m^7^GpppA_m_U. Protein residues involved in the ligand interactions are represented as *sticks* and labeled. Residues from neighboring nsP1 are colored in *white* and residue numbers are *highlighted* and *underlined*. *Dashed lines* indicate polar interactions. Water molecules are highlighted as *gray spheres,* while magnesium as *magenta spheres*. *D*, structure comparison of nsP1(H37A) with Cap-1 RNA (m^7^GpppA_m_U, this work) and Cap-0 RNA (m^7^GpppAU-RNA10, PDB 7FGI (6)). The electrostatic surface is calculated by the PyMol-molecular visualization system. Positive charges are colored in *blue*, neutral in *white*, and negative in *red*. Cap-1 RNA and Cap-0 RNA are shown as *sticks* and colored in *yellow* and *white*, respectively. CHIKV, chikungunya virus; nsP, nonstructural protein.

To reveal the decapping mechanism of nsP1, we determined the capping/decapping inactive mutation H37A nsP1-mut in complex with SAH and mRNA analog, Cap-1 RNA m^7^GpppA_m_U at a resolution of 2.4 Å by cryo-EM (Fig. 4, Table S1 and Fig. S1). The overall structure is similar to that of nsP1 in complex with capping subtract SAH and m^7^GTP (PDB 7FGG, RMSD 1.22 Å (12)). All twelve copies of the GTase active sites in the nsP1 ring are occupied by m^7^GpppA_m_U RNA and SAH (Fig. 4*A*). The guanine base and ribose ring of the m^7^G_0_ cap are located at the same position as that of the previously reported nsP1-m^7^GTP structure (PDB 7FGG (12)) (Fig. 4*B*). The guanine base, bound to the pocket formed by residues D152, Y154, Y248, and E250, forms stacking interactions with the aromatic side chain of Y154 and Y248 and is further stabled by a hydrogen bond (H-bond) with E250. The ribose ring makes distant van der Waals interaction with V243 and forms an H-bond with the side chains of D152 and Y285. Further, the triphosphates of m^7^G_0_ cap form H-bonds with the positively charged side chains of arginine residues, R41 and R70 (Fig. 4*B*). The α- and β-phosphates interact with a magnesium ion, while the γ-phosphate indirectly interacts with a magnesium ion in a water-mediated manner. The m^7^G_0_ cap was captured in a preguanylyl-transferring state, explaining the importance of the magnesium coordination to its α- and β-phosphates at the GTase active site.

The nucleotides A1 and U2 of Cap-1 RNA, stacked on to each other, are both located in the groove between the neighboring nsP1-mut molecules (Fig. 4*C*). The A1 base is stabilized by the T33-P34-N35 loop and V279 of the neighboring nsP1. The A1 base also forms H-bond with the backbone of V279 (neighboring nsP1-mut and water-mediated H-bonds with N35 and D36). The 2′-O-me group of A1 ribose forms van der Waals interaction with T33. The U2 base is sandwiched between the A1 base and the side chain of R289 from the neighboring nsP1(H37A). Another residue E54 from the neighboring nsP1-mut forms polar interaction with it. The phosphate between A1 and U2 forms an H-bond with the side chain of K99. Compared to our reported structure of nsP1-mut in complex with Cap-0 RNA m^7^GpppAU-10 (12), the m^7^G_0_ cap and A1 of m^7^GpppA_m_U are located at a similar position. However, the U2 base is stacked to A1 instead of binding to the distant pocket for the uridine base on the opposite side and facing the periphery of the nsP1 upper ring (Fig. 4*D*). This conformation may be due to the absence of nucleotides after U2, which does not seem to affect the decapping activity.

## Discussion

We discovered that nsP1 possesses a novel decapping activity to remove mGp cap from various sources including short capped RNA and cellular mRNAs, where the resulting mRNAs bearing the 5′end diphosphates in turn will activate RIG-I–mediated immune response (15). By aligning our structure to the previously reported m^7^GTP-bound nsP1 (PDB 7FGG (12)) to model the H37 and m^7^G_0_ interactions, the NƐ-H37 nucleophilic attack on the α-phosphate of m^7^GpppA_m_U to form m^7^Gp-nsP1 covalent intermediate is enabled by: (1) structural rearrangement occurs at the triphosphates moiety of m^7^GpppA_m_U, where (2) the α- and β-phosphates move closer towards H37 (α-phosphate to H37 distance reduced to ∼3.2 Å) coordinated with magnesium ion and water molecules (Fig. 4, *B* and *C*). The orientation differences of the capped-RNA substrate binding to the nsP1 pocket could be due to the short capped RNA presented here. which favors the typical base stacking conformation and is stabilized by neighboring nsP1 residues, R289 and E54 (Fig. 4, *C* and *D*). Overall, nsP1 is able to transfer the m^7^Gp moiety from capped mRNA to the diphosphorylated 5′ end of viral transcript thus generating an authentic cap structure. These findings are consistent with previous work demonstrating that nsP1 can release significant amounts of intermediates of the reaction and can decap RNA substrates (16). They also suggested that capping of the alphaviral RNA most likely occurs simultaneously with transcription prior to folding of the conserved SL1 loop, thereby protecting the viral RNA from being decapped (16).

**Figure 5:**
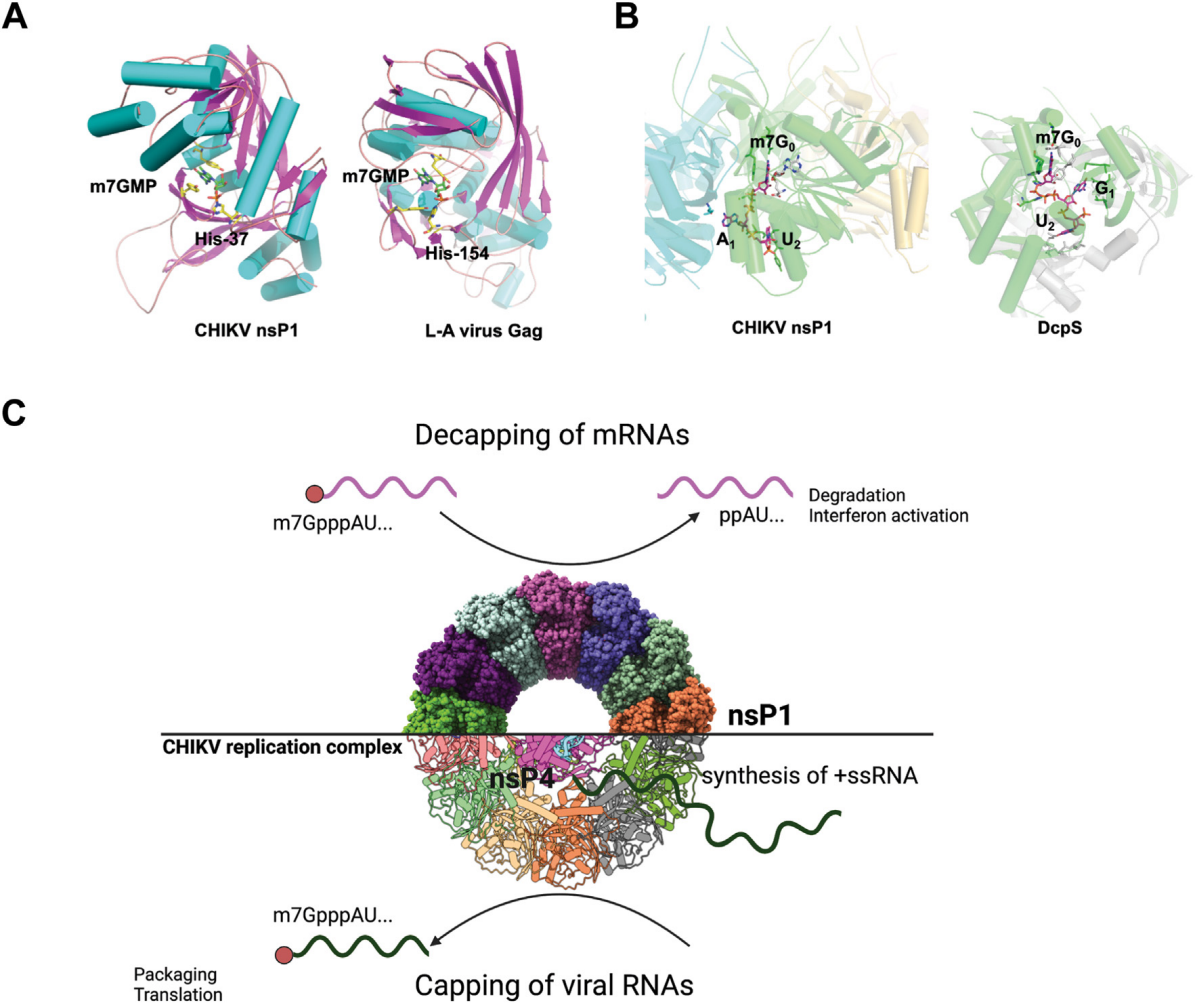
**CHIKV nsP1 structural comparison with other proteins and overview of decapping and capping functions.***A*, the structures are colored by the secondary structures to highlight the similarity between the CHIKV nsP1 and L-A virus Gag monomer (PDB code: 1m1c). The catalytic residues H37 in CHIKV nsP1, H154 in L-A Gag, and the trapped m^7^Gp are shown as *sticks*. *B*, the dimer partner molecule of DcpS is shown in *gray*, which forms part of the m^7^G_0_ binding pocket at the location equivalent to SAH in nsP1 structure. The Cap-0RNA, SAH, and nearby protein residues from nsP1 and DcpS are shown as *sticks* (PDB code: 6trq). *C*, the hypothesis is that nsP1 decapping may occur in the early replication targeting cellular RNAs and switches to viral RNA capping in the presence of +ssRNA synthesis in the replication complex before exporting it into the cytosol for packaging and formation of mature virions (created with BioRender.com). CHIKV, chikungunya virus; DcpS, scavenger decapping enzyme; nsP, nonstructural protein.

The decapping activity of nsP1 has several potential roles including responding to host defense. In this scenario, nsP1 is analogous to the Gag protein of the L-A virus from yeast (Fig. 5*A*) (17, 18, 19, 20, 21, 22) and the PA endonuclease, a subunit of the influenza polymerase from the influenza virus to a lesser extent (23). L-A virus Gag protein decaps cellular mRNAs as a strategy to protect its own uncapped viral transcripts as well as enhance viral mRNA expression by effectively extending its half-life. The success of the virus hijacking host translation machinery depends on L-A virus Gag protein decapping activity, as viral mRNA is not translated without it (10, 24). While cap snatching by PA endonuclease from influenza virus similarly has an active site centered on a histidine residue, cleaves the sequence 10 to 13 nucleotides from the cap structure *via* endonuclease activity (23) resulting in decapped host mRNAs which are targeted for degradation. This strategy to promote cellular mRNA degradation is similarly employed by SARS-CoV-2 nsp1 which is hypothesized to recruit cellular endonuclease to promote RNA cleavage resulting in decreased cellular mRNA translation globally, effectively reducing IFN activation (25). This targeted degradation of cellular mRNAs appears to be key in establishing alphaviral replication in the cell as it has been observed that 5′-3′ exonuclease XRN 1 is essential to SINV infection (26).

As CHIKV nsP1 has some sequence recognition, m^7^GpppA_m_U is preferred over m^7^GpppA_m_G (Fig. 1*D*) unsurprising seeing as capping also demonstrates sequence preference (12). This contrasts with the vaccinia virus decapping enzyme D10 which cannot discriminate between viral and cellular mRNAs, resulting in endonucleolytic cleavage of host mRNAs to restrict gene expression. CHIKV nsP1 may selectively decap viral mRNAs or other cellular mRNAs beginning with nucleotide sequence AUG (16) to prevent translational overload, whereby the capacity of the translation machinery becomes overwhelmed due to an excess of mRNAs produced in early infection. Comparing decapping more broadly with nonviral proteins such as the scavenger decapping enzyme (Fig. 5*B*), a mRNA decapping enzyme that is a member of the histidine triad (HIT) family of hydrolases which is involved in the removal of cap following mRNA decay (27, 28, 29). Similar to CHIKV nsP1, it contains an active site and a nucleotide binding pocket that coordinates the pyrophosphate bond with respect to the catalytic residues and catalyzes the cleavage of m^7^GpppN. However, its activity is strongly dependent on the mRNA body length. CHIKV nsP1 does not appear to have this restriction as it decaps RNAs of twelve nucleotides in length. Interestingly, scavenger decapping enzyme binds more efficiently to cap structures rather than capped RNAs and can compete with eukaryotic translation initiation factor 4E for accessibility, likely as a regulatory mechanism to prevent premature binding of eukaryotic translation initiation factor 4E to degraded mRNAs.

Alternatively, the role of decapping in alphaviruses may also act as a mechanism to regulate virus infection and replication. This may serve as an important signal for the virus to maintain the ratio of capped *versus* uncapped viral RNAs, which is crucial for viral replication, translation, immune escape, and infectivity (30). Moreover, decapping and capping may not be mutually exclusive activities as it is possible to have all twelve active sites functioning synchronously. The capping/decapping event may also occur in a local substrate concentration-dependent manner to meet demands at different stages of the viral replication as our data suggested that high concentrations of capped products (>1 mM) need to be present for decapping to occur *in vitro* (Fig. 1). In addition, uncapped viral RNAs have also been implicated increased Sindbis virus disease (31). The authors attribute the effect to decreased capping, but increased decapping may also be present to promote the formation of uncapped viral genomes resulting in increased pathogenicity. Further work is warranted to investigate the viral and host factors that trigger the switch between nsP1 capping and decapping activities.

Alphaviruses physically protect their own RNA replication intermediates through isolation by replicating in membrane-bound and compartmentalized replication factories, termed spherules that exclude certain protein synthesis factors and host factors. Despite evolving effective means of interfering with the development of the innate immune response, many alphaviruses are known to induce a type I IFN response *in vivo* (32). CHIKV nsP1 decapping activating IFN signaling pathway *via* RIG-I would be a novel mechanism to explain this observation. Previously in Sindbis virus, cells exposed to low doses of IFN for a short period can still be productively infected and support virus replication. Released IFN can either protect the uninfected cells, perform further rounds of more efficient priming, or stimulate IFN-β release and slow down cytopathic effect development. This suggests a fine balance between viral replication and antiviral response in the early stages of infection (32). While this is observed in CHIKV nsP1, this effect may extend to other alphaviruses’ nsP1s as they share conserved sequence homology (Fig. 2*C*). This also has potential implications for alphavirus-based self-amplifying RNAs, which utilize the nsPs as the functional replicase which could restrict self-amplifying RNAs amplification and antigen expression (33). Unexpectedly, we observed some nsP1 activity–independent activation of IFN pathway as signal was not eliminated in the enzymatically inactive nsP1-mut protein, which may be due to other functions of nsP1 including nsP1–host protein interactions, membrane binding, negative-strand synthesis, and more recently, migrasome formation (34, 35, 36, 37) (Figs. 2 and 3).

Combining with the recently elucidated CHIKV replication complex gives insight that the viral replication is tightly protected in the spherule, allowing for ssRNA capping to be orchestrated by nsP1 and nsP2 before exporting it into the cytosol (38, 39) which may reduce the activation of RIG-I–mediated immune response. Spherule-free nsP1-2-4 complexes were found in infected cells, suggesting that some other unknown virus–host interaction may be involved, potentially including nsP1-mediated decapping of viral and host mRNAs. Therefore, we hypothesize that nsP1 decapping may occur in the early replication complex during the initial stages of infection regulating viral genome RNA’s role to serve as a template for replication and to decap cellular mRNAs for targeted degradation (Fig. 5*C*). Subsequently, as the infection progresses resulting in virus-mediated transcription inhibition and host translational shutoff to dampen IFN antiviral response. Finally, the formation of the mature replication complex and synthesis of +ssRNA triggers nsP1 to switch to viral RNA capping before exporting it into the cytosol for the packaging and formation of mature virions.

### Limitations and future work

The main limitation of this method to evaluate RNA decapping activity may not reflect viral infection and replication. It omits the role of the other nsPs and host factors during viral replication after infection and may overemphasize the decapping activity, when decapping might be a rare occurrence in early viral infection, to prevent translational overload, when GTP/SAM is depleted or to maintain the capped/uncapped RNA ratio to regulate the virus replication cycle. Future work may be to systemically dissect nsP1-mediated virus-host integration pathways and their role in viral infection and replication.

## Experimental procedures

### CHIKV nsP1 recombinant protein preparation

WT and mutant nsP1 H37A (hereafter referred to as nsP1-mut) were expressed and purified as described in the study by Zhang *et al* (6).

### RNA 5′ end capping assay

The method previously described by Li *et al*. was used with modifications (40). CHIKV 5′ UTR sequence is used as the template to synthesize the substrate RNA. The 5′ triphosphorylated (ppp) single-stranded 12-mer RNA AUGGCUGCGUGA labeled with a FAM dye was named “pppAU-10 FAM”. A 5′ diphosphorylated (pp) single-stranded 12-mer RNA AUGGCUGCGUGA was labeled with a FAM dye at the 3′ end and named as “ppAU-10 FAM”. A Cap-0 RNA was synthesized based on the above sequence, named as “m^7^GpppAU-10” labeled with FAM dye or Alexa 647 was named “m^7^GpppAU-10 FAM” and “m^7^GpppAU-10 Alexa 647,” respectively. The RNAs were synthesized by Trilink Biotechnologies and Bio-Synthesis Inc. The capping reaction is in two steps, first, a covalent m^7^Gp-nsP1 intermediate reaction was prepared in a 20 μl containing 20 μM nsP1, 50 mM Tris–HCl pH 7.5, 2 mM DTT, 10 mM KCl, and 2 mM MgCl_2_ and 0.5 mM SAM and 1 mM GTP or an alternative substrate as cap donor was incubated at 30 °C for 2 h. Second, after the preparation of the covalent m^7^Gp-nsP1 is used for the transfer to RNA recipient in a 20 μl mixture containing 5 μl covalent intermediate, 50 mM Tris–HCl pH 7.5, 2 mM DTT, 2 mM MgCl_2_, and 1 μM synthetic RNA, and 20 U Murine RNase Inhibitor (New England Biolabs). The reactions were incubated at 30 °C for 12 h and terminated by adding the stop solution 2× RNA loading dye (95% formamide, 0.02% bromophenol blue, 0.01% xylene cyanol, 0.02% SDS, 1 mM EDTA). The capped RNA products were separated by 20% denaturing RNA 8M urea PAGE gel and visualized using ChemiDoc MP imaging system (Bio-Rad). The bands were quantified with Image Lab (Bio-Rad; https://www.bio-rad.com/en-sg/product/image-lab-software?ID=KRE6P5E8Z) and plotted and analyzed on Prism 9.3.1 (GraphPad; https://www.graphpad.com/).

### Mammalian total RNA and mRNA extract preparation

Expi293 cells in suspension were grown, pellet at 200*g* for 5 min at 4 °C and the supernatant was removed. The pellet was resuspended in PBS and incubated with TRIzol reagent (Thermo Fisher Scientific). Chloroform was added and the mixture was spun at 12,000*g* for 30 min at 4 °C to separate the aqueous and organic phase. Total RNA was extracted from the aqueous phase after the addition of isopropanol and washing with ethanol. The pellet is spun for an additional 10 min at 7,500*g* at 4 °C, the ethanol was removed and air-dried. The pellet is resuspended in the desired amount of RNase-free water (Hyclone), and the concentration is determined by spectrophotometry. The RNA sample is denatured at 65 °C with RNA loading dye and run on a 0.6% agarose gel at 100 V for 1 h for visualization. For mRNA extracts, total RNAs are pooled and further purified using the Oligotex mRNA kit (Qiagen) as per the manufacturer’s instructions.

### Immunoblotting of CHIKV nsP1 guanylylation reaction

Reactions were prepared in 20 μl containing 1.5 μM nsP1, 50 mM Tris–HCl pH 7.5, 2 mM DTT, 10 mM KCl, and 2 mM MgCl_2_ and 0.5 mM SAM and 1 mM GTP or alternative cap donors such as mRNA or total RNA extracts and incubated for 2 h at 30 °C. It was stopped with the addition of Laemmli sample buffer, and products were loaded onto a 12% SDS-PAGE gel. The 12% SDS-PAGE gel was stained with Coomassie blue or prepared for immunoblotting and transferred onto a polyvinylidene difluoride membrane (Sigma-Aldrich) with 1× Transfer buffer (48 mM Tris 39 mM glycine and 20% methanol) by Trans-Blot Turbo (Bio-Rad) for 20 min at max 2.5 A and 25 V. The membrane was blocked with 5% bovine serum albumin 1× PBS for 30 min at room temperature and subsequently incubated with the primary antibody anti-7-m^7^G cap (RN016M, MBL International Corporation) 1 μg/ml overnight at 4 °C and washed thrice with washing buffer 1% bovine serum albumin 1× PBS 0.1% Tween-20 for 5 min with agitation. The secondary antibody (anti-mouse IgG horseradish peroxidase-linked #7076, Cell Signalling Technology) is added to the membrane for 1 h at room temperature and washed thrice again with washing buffer before the addition of the chemiluminescence reagent (Immobilon Western Chemiluminescent horseradish peroxidase Substrate, Millipore). The extra reagent is removed from the membrane and visualized on the ChemiDoc MP imaging system (Bio-Rad).

### Mammalian total RNA treatment with recombinant CHIKV nsP1 and alkaline phosphatase

To generate dephosphorylated 5′ RNA, total RNA was treated with alkaline phosphatase (Promega) 100 μl reaction was set up as per manufacturer’s instructions. To generate CHIKV nsP1-decapped RNA, recombinant CHIKV nsP1 treated total RNA, 100 μl reaction was set up with 30 mg total RNA added to 20 μM recombinant CHIKV nsP1 in 50 mM Tris–HCl pH 7.5, 2 mM DTT, 10 mM KCl, and 2 mM MgCl_2_ incubated at 30 °C for 2 h. Subsequently, the reactions were then incubated with TRIzol reagent (Thermo Fisher Scientific). Chloroform was added and the mixture was spun at 12,000*g* for 30 min at 4 °C to separate the aqueous and organic phase. Total RNA was extracted from the aqueous phase after the addition of isopropanol and washing with ethanol. The pellet is spun for an additional 10 min at 7500*g* at 4 °C, the ethanol was removed and air-dried. The pellet is resuspended in the desired amount of RNase-free water (Hyclone), and the concentration is determined by spectrophotometry. The RNA sample is denatured at 65 °C with RNA loading dye and run on a 0.6% agarose gel at 100 V for 1 h for visualisation.

### *In vitro* transcription of RNA 3p10LA9

RNA 3p10LA9 pppGGAUUUCCACCUUCGGGGGAAAUCC were transcribed *in vitro* using T7 RNA polymerase as described previously (14, 41). The reactions contain 40 mm Hepes pH 7.5, 30 mm MgCl_2_, 2 mm spermidine, 10 mm DTT, 0.01% Triton X-100, 5 mm GTP, and 4 mm NTP (CTP, ATP, and UTP), 1 μm DNA template (IDT), 400 to 600 nm T7 RNA polymerase, 0.2 U·mL^−1^ thermostable inorganic pyrophosphatase (New England Biolabs) for overnight at 37 °C. Phenol:chloroform:isoamyl alcohol (25: 24:1) (Merck) was used to stop *in vitro* transcription reactions and extract RNA. The RNA was precipitated overnight at −80 °C by adding three volumes of ethanol 95% in the presence of 0.1% (v/v) of sodium acetate. The RNAs were further isolated by Hi-Trap Q HP column and excised from 20% denaturing urea-PAGE. The quality of expected RNAs was determined again on 20% denaturing urea-PAGE prior to downstream experiments.

### Alphavirus nsP1 activity and its mutants in A549 Dual and A549 Dual KO-RIG-I

A549 Dual and A549 Dual KO-RIG-I cells (InvivoGen) were maintained in Dulbecco’s Modified Eagle Medium (Gibco) containing 10% fetal bovine serum, 100 U/ml penicillin, 100 μg/ml streptomycin at 37 °C in a 5% CO_2_ incubator. A549 Dual and its KO-RIG-I equivalent cells express secreted Lucia luciferase reporter gene under the control of an ISG54 minimal promoter in conjunction with five IFN-stimulated response elements. Both cell lines were grown on 96-well plates and were first transfected with CHIKV nsP1 expression plasmid (pCMV-CHIKV nsP1) or its catalytically inactive mutant H37A (nsP1-mut) by PEI MAX (Polysciences) for transient expression for 24 h. Subsequently, the supernatant was collected, and luciferase activity is measured by QUANTI-Luc reagent (as per manufacturer’s instructions) onto a 96-well plate (Corning) with a microplate reader (Synergy H1 BioTek). The luciferase activity is normalized to cells transfected with a control vector pBad His6 MBP TEV LIC (Addgene #37503) and cells that were not transfected. Each experiment was performed in triplicate and replicated. The results were analyzed with an unpaired *t* test on Prism 9.3.1 (GraphPad). The experiment was performed similarly with VEEV nsP1 and its inactive H37A (VEEV nsP1-mut) mutant as well as SINV nsP1 and its inactive H39A (SINV nsP1-mut) mutant.

### CHIKV nsP1–treated RNA and alkaline phosphatase–treated RNA in A549 Dual and A549 Dual KO-RIG-I

Both cell lines were grown on 96-well plates were first transfected with 100 ng of treated RNA with LyoVec (InvivoGen). After 48 h, the supernatant was collected, and luciferase activity is measured by QUANTI-Luc reagent (as per manufacturer’s instructions) onto a 96-well plate (Corning) with a microplate reader (Synergy H1 BioTek). The controls were cell lines transfected with untreated total RNA and hairpin RNA 3p10LA9. The results were analyzed with an unpaired *t* test on Prism 9.3.1 (GraphPad).

### Lentivirus and vector production

The CHIKV nsP1 WT and H37A mutant genes were cloned into the vector pInducer20 (Addgene #44012), pInducer CHIKV nsP1, and pInducer CHIKV nsP1-mut, respectively. Lentiviral supernatants were generated by transfection of HEK293T cells according to Mirus Bio's TransIT transfection protocols and pooled 96 h after transfection. Lentiviral titers were measured with Lenti-X GoStix Plus (Takara Bio) and concentrated with Lenti-X concentrator as per the manufacturer’s instructions. The lentivirus was resuspended in PBS and stored at −80 °C until required.

### A549 Dual and A549 Dual KO-RIG-I tet-inducible CHIKV nsP1 expression cell lines

A549 Dual and A549 Dual KO-RIG-I cells (InvivoGen) were transduced with pInducer CHIKV nsP1 and pInducer CHIKV nsP1-mut lentivirus in appropriate media containing 8 μg/ml polybrene (Sigma-Aldrich). Media was replaced after 24 h and at 48 h selected with geneticin G418 (Gibco) and 1 μg/ml doxycycline was added for induction of protein expression. CHIKV nsP1 and CHIKV nsP1-mut expression was verified by Western blot (Fig. 3*D*). A549 Dual CHIKV nsP1, A549 Dual KO-RIG-I CHIKV nsP1, A549 Dual CHIKV nsP1-mut, and A549 Dual KO-RIG-I CHIKV nsP1-mut were grown on 24-well plates. Cells were washed with 1× PBS (Gibco), and media was replaced with Dulbecco’s Modified Eagle Medium (Gibco) containing 10% tet-system fetal bovine serum and 1 μg/ml doxycycline. After 5 min and 72 h, the supernatant was collected, and luciferase activity is measured by QUANTI-Luc reagent (as per manufacturer’s instructions) onto a 96-well plate (Corning) with a microplate reader (Synergy H1 BioTek). Each experiment was performed in triplicate and the results were analyzed with an unpaired *t* test on Prism 9.3.1 (GraphPad).

### Cryo-EM grid preparation and microscopy

SAH and m^7^GpppA_m_U were incubated with nsP1-mut under 4°C overnight before preparing Cryo-EM grids. The carbon side of the Quantifoil R1.2/1.3 gold 300 mesh grid was covered with one layer of graphene following a published protocol (42). After glow-discharging for 10 s at low energy with Plasma cleaner, a 3 μl sample was applied to the grids, blotted for 2.5 s with blot force −2, and plunge-frozen in liquid ethane using Vitrobot (Thermo Fisher Scientific). Cryo-EM grids were imaged on a Thermo Fisher Scientific 300 kV TEM Titan Krios using Gatan K2 direct electron detector or Falcon III detector. The detailed information of data collection was summarized in Table S1.

### Cryo-EM image processing and model building

The collected movies of different samples were motion-corrected and dose-weighted using MotionCor2 (https://emcore.ucsf.edu/ucsf-software) (43). The output micrographs were imported to CryoSPARC v3.2.0 (https://cryosparc.com/) (44). After contrast transfer function (CTF) estimation, micrographs with CTF-estimated maximum resolution better than 4 Å were selected to pick particles using Template Picker. After one round of 2D classification, the selected good particles were subjected to heterogeneous refinement using the previously published map of CHIKV nsP1 as an initial model. After several rounds of heterogeneous refinement, the best classes of different samples were performed one round of homogeneous refinement with no symmetry (C1) and resulted in 3.34 Å maps. After CTF refinement and one round of nonuniform refinement with C12 symmetry, the maps were improved to 2.41 Å. The maps were further sharped with a B-factor of −50. Figures representing the map features were prepared with UCSF Chimera (45). The details of the data collection and processing refer to the extended data Fig. S1 and Table S1.

Deposited nsP1 structure (PDB: 6Z0V (5)) was used as a template for model building and refinement. The model was further improved through cycles of real-space refinement (with Ramachandran restraints and secondary structure restraints) in Phenix and following manual corrections by Coot (46). The refinement statistics of the model are summarized in Table S1. Figures representing the structural features were prepared with UCSF Chimera (45) and PyMOL (http://pymol.org). The H37 was manually modeled into the current structure by aligning to template m^7^GTP-bound WT nsP1 (PDB 7FGG (12)).

## Data availability

The cryo-EM density map for the CHIKV nsP1(H37A) in complex with SAH and m^7^GpppA_m_U has been deposited in EM Database under the accession code EMD-36159. The corresponding atomic coordinates have been deposited in the Protein Data Bank under accession code 8JCE.

## Conflict of interest

The authors declare that they have no conflict interests with the contents of this article.
