# Supplementary material for: Chikungunya virus nonstructural protein 1 is a versatile RNA capping and decapping enzyme
Source: J Biol Chem. 2023 Oct 31;299(12):105415. doi: 10.1016/j.jbc.2023.105415 (PMC10687048; doi:10.1016/j.jbc.2023.105415)

**Supplemental Materials**

**Supplemental data**

**Table S1. Cryo-EM data collection, refinement and validation statistics.**

| **Data collection and processing** | |
| --- | --- |
| EM equipment | Titan Krios (Thermo Fisher Scientific) |
| Voltage (kV) | 300 |
| Detector | Gatan K2 |
| Pixel size (Å) | 0.85 |
| Defocus range (µm) | -0.6 ~-3.5 |
| Magnification | 165,000 × |
| Frames | 40 |
| Total dose (*e^−^* /Å^2^) | 60 |
| Number of collected micrographs | 6,597 |
| Number of selected micrographs | 6,111 |
| Number of used particles | 70,128 |
| Map Resolution (Å) | 2.41 |
| Symmetry | C12 |
| Map sharpening B-factor (Å^2^) | 50 |
| **Refinement** | |
| Initial model | 7DOP |
| Model Resolution (Å) | 2.6 |
| Model composition | |
| Chains | 12 |
| Non-hydrogen | 44304 |
| Residues | Protein: 5448 Nucleotide: 0 |
| Water | 588 |
| Ligands | ZN: 12  MG:12  YG4:12 |
| B factors (Å^2^) | |
| Protein | 46.34/144.72/71.22* |
| Ligand | 73.01/97.20/73.94* |
| Water | 50.13/79.33/62.75* |
| R.m.s. deviations | |
| Bonds (RMSD) | 0.003 |
| Bonds length (Å) | 0.545 |
| Validation | |
| MolProbity | 1.05 |
| Clashscore | 2.67 |
| Ramachandran plot statistics (%) | |
| Preferred | 98.11 |
| Allowed | 1.89 |
| Outlier | 0.00 |

*Minimum/maximum/mean

**Fig S1. Cryo-EM analysis of nsP1.**

**(A-D)** FSC curves, the viewing direction distribution plot, local resolution map and model-to-map FSC of different nsP1 samples. **(E)** Flowchart for cryo-EM data processing. **(F)** Density map of SAH, m^7^GpppA_m_U and the surrounding residues (σ=7).


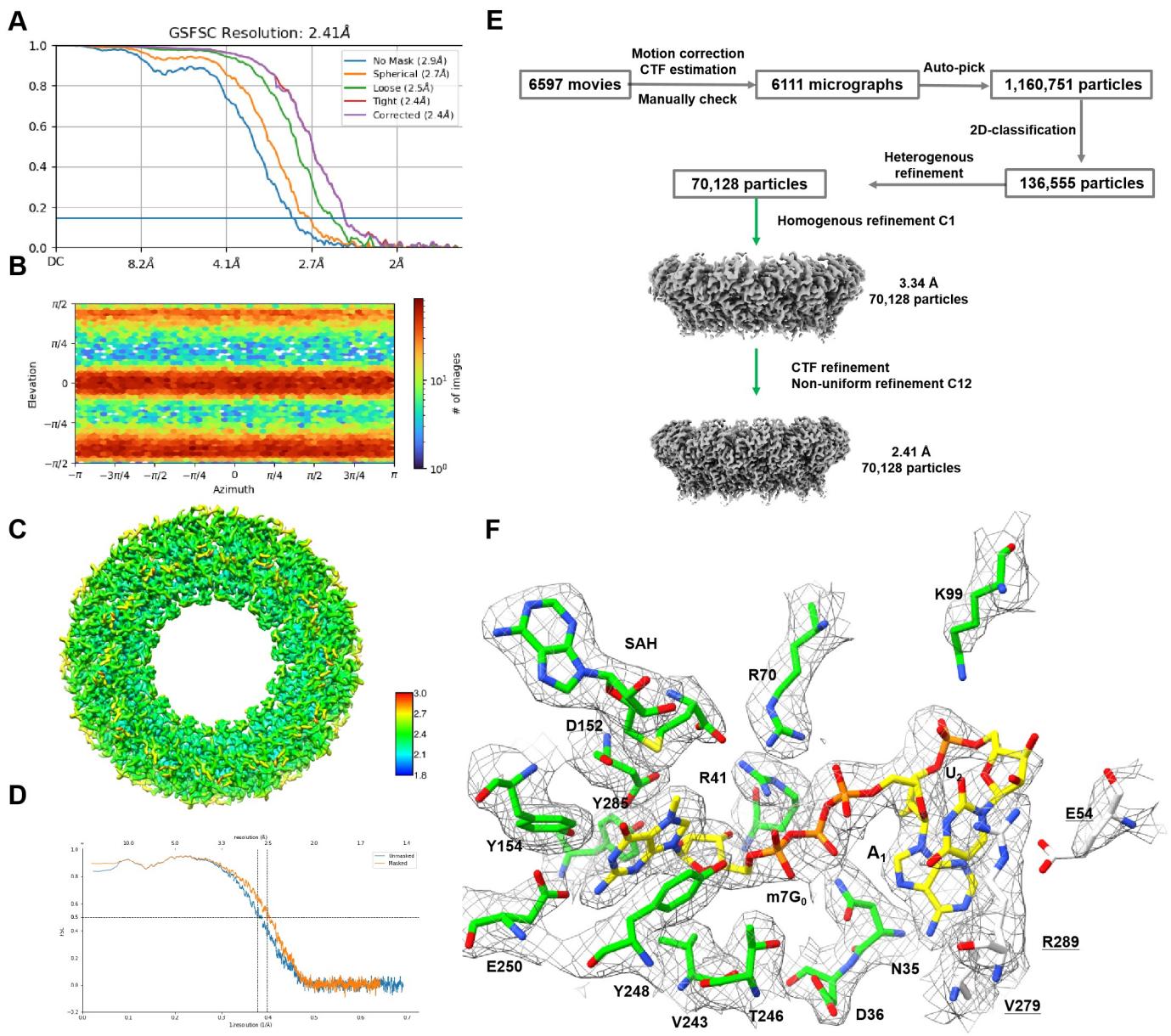

Supplement: Supplemental data [file mmc1.docx]
